# Genomic epidemiology of *Neisseria gonorrhoeae* in Shenzhen, China, during 2019–2020: increased spread of ceftriaxone-resistant isolates brings insights for strengthening public health responses

**DOI:** 10.1128/spectrum.01728-23

**Published:** 2023-09-21

**Authors:** Di Wang, Yamei Li, Chi Zhang, Yaling Zeng, Junping Peng, Feng Wang

**Affiliations:** 1 NHC Key Laboratory of Systems Biology of Pathogens, Institute of Pathogen Biology, Chinese Academy of Medical Sciences & Peking Union Medical College, Beijing, China; 2 Shenzhen Center for Chronic Disease Control, Shenzhen Institute of Dermatology, Shenzhen, People’s Republic of China, China; 3 Key Laboratory of Respiratory Disease Pathogenomics, Chinese Academy of Medical Sciences & Peking Union Medical College, Beijing, China; 4 Key Laboratory of Pathogen Infection Prevention and Control (Peking Union Medical College), Ministry of Education, Beijing, China; 5 State Key Laboratory of Respiratory Health and Multimorbidity, Chinese Academy of Medical Sciences, Beijing, China; Yale University, New Haven, Connecticut, USA

**Keywords:** molecular surveillance, antimicrobial resistance, multilocus sequence typing, *N. gonorrhoeae *sequence typing for antimicrobial resistance, whole-genome sequencing

## Abstract

The antimicrobial resistance (AMR) in gonorrhea poses global threat of increasing public health concern. In response to this concern, molecular surveillance has been widely utilized to detail the changes in the evolution and distribution of *Neisseria gonorrhoeae* during AMR transmission. In this study, we performed a comprehensive molecular surveillance of 664 *N*. *gonorrhoeae* isolates collected in Shenzhen, one of the cities with the largest mobile population in China, 2019–2020. In 2020, ceftriaxone showed an unprecedented high resistance rate of 24.87%, and 67.83% of the ceftriaxone-resistant (Cro-R) isolates harbored a nonmosaic *penA* allele. The Cro-R isolates with nonmosaic *penA* alleles showed a tremendous increasing trend from 0.00% in 2014 to 20.45% in 2020, which proves the need for monitoring nonmosaic *penA*-related resistance. Importantly, genotyping indicated that multilocus sequence typing ST11231 (35.71%) had a notable rate of ceftriaxone resistance, which might become the focus of future surveillance. Whole-genome sequencing analysis showed that the internationally spreading FC428 clones have circulated in Shenzhen region with typical ceftriaxone resistance (MIC ≥ 0.5 mg/L) maintained. Our surveillance combined with genomic analysis provides current information to update gonorrhea management guidelines and emphasizes that continuous AMR surveillance for *N. gonorrhoeae* is essential.

**IMPORTANCE:**

We conducted a comprehensive molecular epidemiology analysis for antimicrobial-resistant *Neisseria gonorrhoeae* in Shenzhen during 2019–2020, which provided important data for personalized treatment and adjustment of monitoring strategy. Briefly, the proportion of ceftriaxone-resistant (Cro-R) isolates reached a stunning prevalence rate of 24.87% in 2020. A typical increment of Cro-R isolates with nonmosaic *penA* alleles proves the necessity of monitoring nonmosaic AMR mechanism and involving it into developing molecular detection methods. Whole-genome sequencing analysis showed that the international spreading FC428 clone has been circulating in Shenzhen with typical ceftriaxone resistance (MIC ≥ 0.5 mg/L) maintained. In summary, we conducted a comprehensive epidemiology study, providing significant data for therapy management. Our results not only improve the understanding of the distribution and transmission of AMR in *N. gonorrhoeae* but also provide effective AMR data for improving surveillance strategies in China.

## INTRODUCTION

Gonorrhea, an infection caused by *Neisseria gonorrhoeae*, is a common global sexually transmitted disease, with an estimated 82 million annual cases (https://www.who.int/teams/immunization-vaccines-and-biologicals/diseases/neisseria-gonorrhoeae). Antimicrobial resistance (AMR) has developed for most antimicrobials in the past few decades and has become a growing global public health burden that requires global monitoring and intervention (1). Excessive use and/or misuse of antimicrobials, limitations in surveillance and control of AMR, and lagging updates of guidelines will drive *N. gonorrhoeae* to acquire and develop AMR (2). *N. gonorrhoeae* has been included in “priority pathogens” by the WHO since 2017 due to the emergence of multi-drug-resistant isolates and rapid growth of antimicrobial resistance rate (3).

It has been proposed to undertake multiple prospective studies on slow-growing resistance to combat widespread AMR in *N. gonorrhoeae*; these include pharmacokinetics/pharmacodynamics (PK/PD) analyses to predict which antimicrobial has the highest likelihood of successful treatment of gonorrhea (4). Another mathematical model aims to repurpose “abandoned” antimicrobial through calculating fitness benefit/cost (5). These promising strategies require AMR surveillance data, reinforcing the importance and urgency for comprehensive surveillance. Nevertheless, the actual AMR distribution of *N. gonorrhoeae* remains vague in most settings because phenotype surveillance produces gaps in AMR surveillance, which is mainly caused by the limited availability of culture methods (6). Moreover, resistant-related isolates with novel *penA* alleles are constantly emerging (7), and therefore, timely surveillance is the only approach to ensure that local guidelines match the actual mode of AMR in *N. gonorrhoeae* (6).

Notably, molecular surveillance fills the gaps in interpretability and comparability, as well as significant differences in the methodology from several regions. Genetic analysis was the most popular molecular analysis for tracking AMR in *N. gonorrhoeae*, including whole-genome sequencing (WGS), multilocus sequence typing (MLST), and *N. gonorrhoeae* sequence typing for antimicrobial resistance (NG-STAR) methods.

Globally, *N. gonorrhoeae* MLST ST1901, ST1903, and ST7363 are the most prevalent MLST genotypes among cefixime-resistant and ceftriaxone-resistant (Cro-R) *N. gonorrhoeae* strains (8, 9). In our previous study, ST7363 was predicted to have the potential to become the next internationally spreading Cro-R sequence type after ST1901 in Shenzhen (10). Another resistance clone of international concern is the FC428 clone (MLST ST1903 and NG-STAR ST233), which has circulated worldwide since it was first reported in Japan (11). Given its typical resistance phenotype, this clone has become the focus of global epidemiological investigations (12).

The rapidly increasing cases of gonorrhea in China place a heavy burden on the country’s healthcare system; in 2021, 127,803 new cases of gonorrhea were reported (http://www.nhc.gov.cn/). However, the incidence of gonorrhea is most likely highly underestimated in China, due to the suboptimal diagnostics, screening, reporting, and epidemiological surveillance performed in many settings. Luo et al. reported an overall prevalence of 0.17% (95% CI, 0.11%–0.28%) for gonorrhea in Shenzhen in 2018 (13), which showed 50% higher than the national rates (1999–2000) reported in 2003 (14). Based on the current situation, high-risk areas should be prioritized while improving the national monitoring capacity. Shenzhen, an economic zone in southern coastal China, has the highest incidence and spread of gonorrhea, given its rapid economic growth, increasing active migrant population, and the permanent residents’ average age is only 32.5 years (15). The special demographic characteristics of highly developed economies make Shenzhen a likely hotspot for gonorrhea, making the region a focus for national surveillance. Therefore, it is vital to monitor AMR in gonorrhea in the Shenzhen region, which would be of great benefit to the national surveillance strategy.

Previously, we performed a comprehensive epidemiological study and explored the correlation between AMR and specific STs using 909 gonococcal isolates in Shenzhen from 2014 to 2018, which provides important data for studies of molecular epidemiology of AMR in *N. gonorrhoeae* (10). Briefly, the increase in ceftriaxone resistance was observed from 0.00% in 2014 and 2016 to 3.62% in 2018 (10); however, the data are limited. Therefore, in this study, to investigate whether this increment would become a potential threat, we performed a follow-up AMR surveillance of 664 *N*. *gonorrhoeae* isolates collected from Shenzhen in 2019–2020 using a genome analysis. Seven AMR alleles in NG-STAR and seven housekeeping alleles in MLST were extracted from the WGS data using bioinformatics method and were used to investigate the evolution and variation of AMR and further identify the distribution of AMR in *N. gonorrhoeae*. Maximum-likelihood method was used to infer what the phylogenetic tree of 664 gonococcal isolates looks like. Overall, early and regular surveillance of Shenzhen, one of the areas with the highest prevalence of gonorrhea in China, can slow the spread of AMR in *N. gonorrhoeae* and allow time for the development of novel antimicrobials and vaccines. Notably, this is the first large-scale, WGS-based, follow-up molecular epidemiology study in the Shenzhen region that may be helpful for gonococcal AMR monitoring strategies and updating gonorrhea management guidelines.

## RESULTS

A total of 664 *N*. *gonorrhoeae* isolates were collected (specifically 270 and 394 isolates in 2019 and 2020, respectively). Isolates were obtained from outpatients with gonorrhea who attended the Shenzhen Center for Chronic Disease Control from 2019 to 2020. No restrictions regarding age, gender, partner, and other behaviors of the patients were considered.

### Demographic characteristics and antimicrobial susceptibility

Based on the demographic characteristics, 591 (89.01%) infections occurred in males with a median age of 30 years. Most patients (656, 98.80%) were heterosexually oriented, and 97.59% of the patients indicated having no previous gonorrhea infections. However, the proportion of patients with symptoms of abnormal discharge showed a significant decrease from 98.68% (897/909, 2014–2018) to 82.23% (546/664, 2019–2020) (chi-square test, *P* = 0.000) (10), indicating a possible increased missed diagnosis rate due to lack of obvious symptoms. The demographic characteristics of the isolates are listed in Table S1.

**Fig 1:**
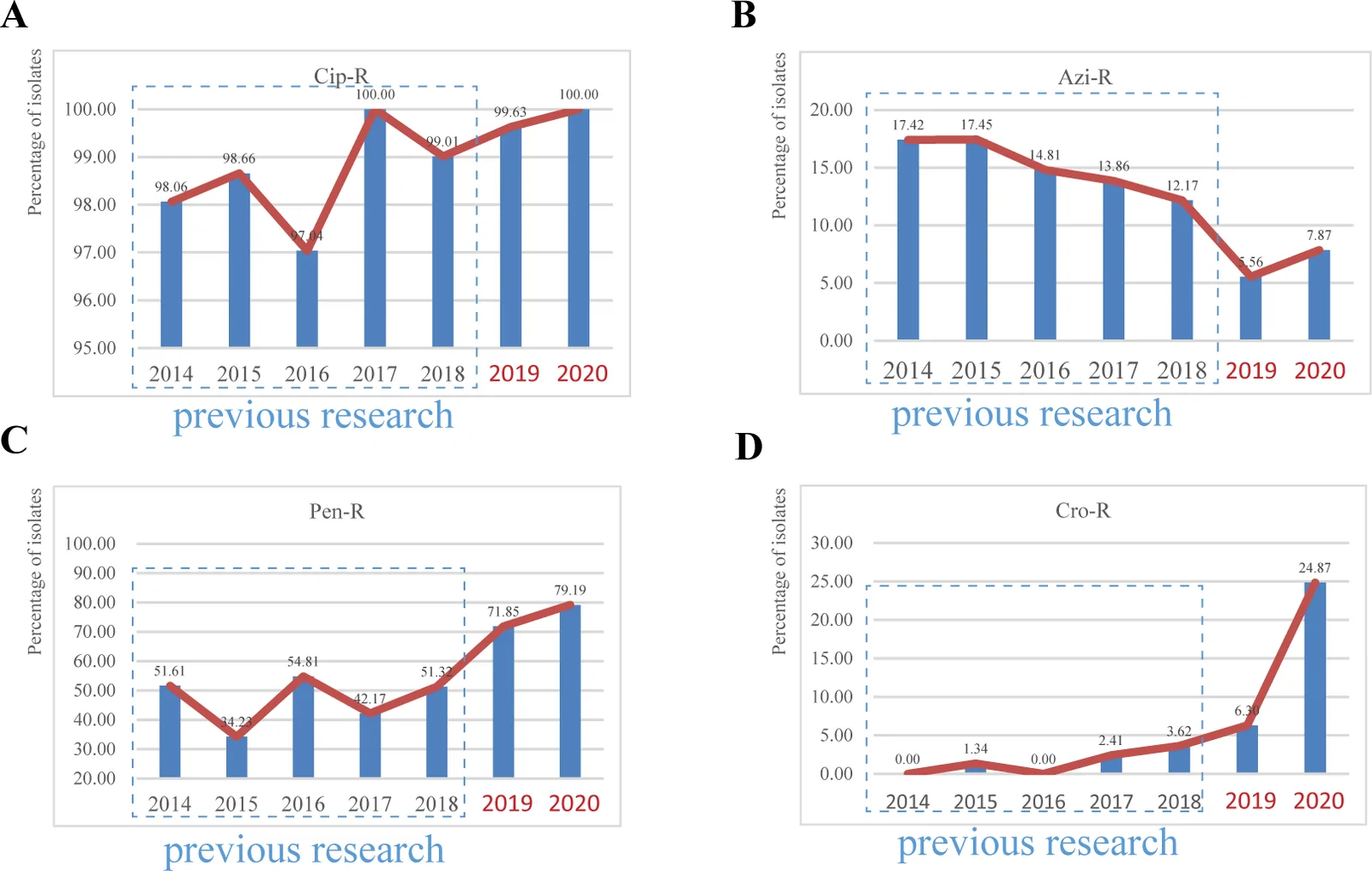
Major antimicrobial susceptibility of *N. gonorrhoeae* in Shenzhen, China, 2014–2020. Percentages of gonococcal isolates resistant to (**A**) Cip, (**B**) Azi, (**C**) Pen, and (**D**) Cro. Bars in dashes boxes were data from our previous research in Shenzhen for 2014–2018. Cip-R, resistant to ciprofloxacin; Azi-R, resistant to azithromycin; Pen-R, resistant to penicillin; Cro-R, resistant to ceftriaxone.

Antimicrobial susceptibility testing was performed for 664 *N. gonorrhoeae* isolates. All 664 *N. gonorrhoeae* isolates were susceptible to spectinomycin (Spec-S), and ciprofloxacin-resistant (Cip-R) isolates remained stable and high since 2014 (Fig. 1A) (10). Azithromycin-resistant (Azi-R) isolates showed a decrease from 12.17% in 2018 to 5.56% in 2019, followed by a slight increase to 7.87% in 2020 (Fig. 1B). Penicillin resistance (Pen-R) showed a steady rise (Fig. 1C). The prevalence rate of ceftriaxone resistance increased from 0.00% in 2014 to 6.30% in 2019, reaching a peak of 24.87% in 2020 (Fig. 1D). The rapid increase in ceftriaxone resistance suggests that the effect of ceftriaxone is powerful threated in Shenzhen that requires an urgent novel therapy. In addition, ST2473, ST1143, ST233, and ST506 range for the major types in the isolates possessing high ceftriaxone MIC values (Fig. S1).

### Molecular typing with MLST

A total of 73 MLST STs were identified, of which 11 were new STs to the database (Fig. S2A). The most common (top five) STs were ST8123 (92/664, 13.86%), followed by ST7363 (77/664, 11.60%), ST7360 (61/664, 9.19%), ST7365 (53/664, 7.98%), and ST1901 (45/664, 6.78%) (Fig. S2A). Compared to previous surveillance in Shenzhen from 2014 to 2018, among all major STs, the ST8123 (9.79% → 13.86%), ST7363 (9.35% → 11.60%), ST7360 (6.05% → 9.19%), and ST7365 (6.16% → 7.98%) increased slightly. Conversely, ST1901 (8.36% → 6.78%) and ST1600 (5.94% → 3.16%) showed a decreasing trend, suggesting that the fitness cost of the ST1901 and ST1600 clones may become higher for continued propagation in the Shenzhen region. ST1901 is widely associated with Cro-DS worldwide (4, 16), and ST1600 has been frequently reported in China because of general ceftriaxone resistance (MIC ≥ 0.75 mg/L) (12, 17). It is worth noting that ST11231, ST7365, and ST8123 showed a notable rate of Cro-R [35.71% (5/14), 28.30% (15/53), and 13.04% (12/92)] in 2019–2020, whereas these STs showed 100% susceptibility to ceftriaxone in 2014–2018. Besides, Cro-R ration of the predominant ST1901 showed significant increment in this study (5.26% → 15.56%) compared to previous AMR surveillance (10). The predominant ST types from 2019 to 2020 are listed in Table 1.

**TABLE 1:** The detailed information of molecular typing of 664 isolates from Shenzhen in 2019–2020

Year	No. of isolates with mosaic *penA* types (%)	Major mosaic *penA* types (no. of isolates)	No. of *penA* types	Major *penA* types (no. of isolates)	No. of NG-STAR STs	Major NG-STAR STs (no. of isolates)	No. of MLST STs	Major MLST STs (no. of isolates)
2019 (270)	62(22.96%)	10.001(26) 34.007(18) 10.003(10)	32	13.001(47) 21.001(34) 10.001(26) 18.001(23) 5.002(22) 34.007(18) 13.002(17) 12.001(13) 10.003(10)	128	495(18) 506(14) 348(11) 2477(9) 1704(7) 501(7) 1102(7) 497(7)	46	7363(40) 7360(35) 8123(25) 1901(24) 7365(17) 9899(14) 1600(11) 7827(10)
2020 (394)	81(20.56%)	34.007(31) 10.001(18) 60.001(14) 10.003(8)	39	12.001(54) 18.001(48) 13.001(44) 13.002(40) 34.007(31) 5.002(23) 21.001(22) 10.001(18) 2.002(17) 103.002(15) 60.001(14) 43.002(11)	202	2473(27) 2477(14) 497(13) 1707(12) 2746(8) 1463(7)	57	8123(67) 7363(37) 7365(36) 7360(26) 1901(21) 10314(18) 1903(13) 7822(12) 1583(11) 7367(11) 7827(11) 1600(10) 1928(10) 15251(10)
Total (664)	143(21.54%)	34.007(49) 10.001(44) 10.003(18) 60.001(15)	51	13.001(91) 18.001(71) 12.001(67) 13.002(57) 21.001(56) 34.007(49) 5.002(45) 10.001(44)	281	2473(33) 2477(23) 497(20) 495(18) 506(17) 1707(16) 348(13) 199(10) 1148(10) 1463(10)	73	8123(92) 7363(77) 7360(61) 7365(53) 1901(45) 10314(27) 7827(21) 7822(21) 1600(21)

**Fig 2:**
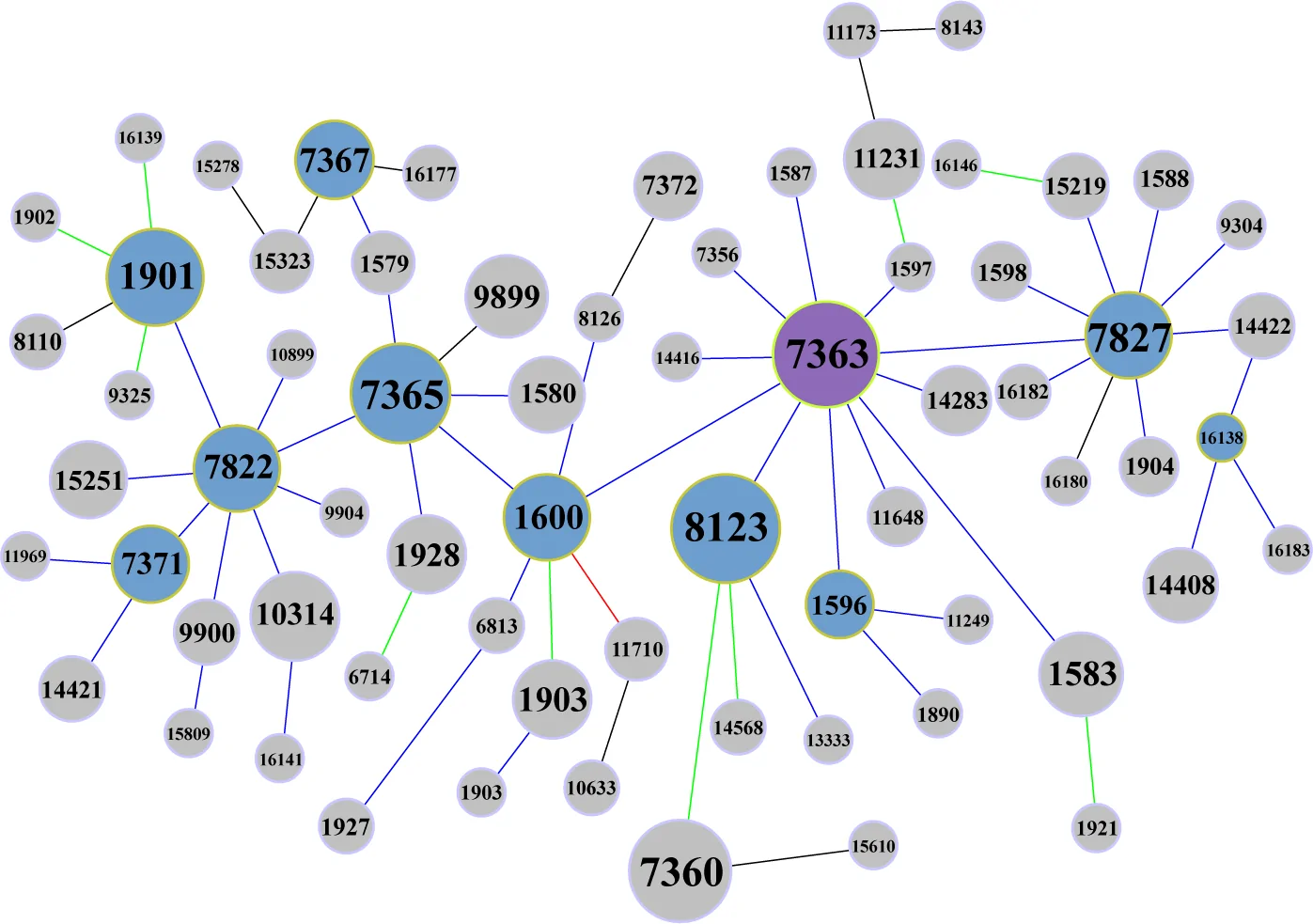
Group 0 of goeBURST analysis. The frequency of occurrence is represented by the font size of MLST STs and the size of the nodes. Node color: purple, group founder; desaturated blue, subgroup founder; light gray, common node. Link color: black, link drawn without recourse to tiebreak rules; blue, link drawn using tiebreak rule 1 (number of SLVs); green, link drawn using tiebreak rule 2 (number of DLVs); red, link drawn using tiebreak rule 3 (number of TLVs).

GoeBURST provides the ability to create groups of closely related strains from MLST data, which presumes that a clonal complex (lineage) is founded by a founder genotype, and genetic variants of that founder reflect the progressive accumulation of additional variations over time (18). The goeBURST divided the 73 MLST STs into 6 groups in this study (Table S2). Most isolates (655/664) were found in group 0. The group founder changed into the ESC-resistant-related ST7363 in Shenzhen for 2019 to 2020 from the local prevalent genotype ST7827 in Shenzhen for 2014 to 2018, using the goeBURST algorithm. Through a logistic regression analysis, ST7363 was found to be closely related to increased MIC of ceftriaxone and was predicted to serve as a reservoir for resistance-related STs (10). Fig. 2 shows that ST7827, ST8123, ST7365, and ST1901 are the subgroup founders with a high-frequency occurrence. Compared to previous surveillance (10), the number of subgroup founders typically decreased (17 → 10), which indicated that the genome complex during 2019–2020 showed a lower diversity.

### Molecular typing with NG-STAR

**Fig 3:**
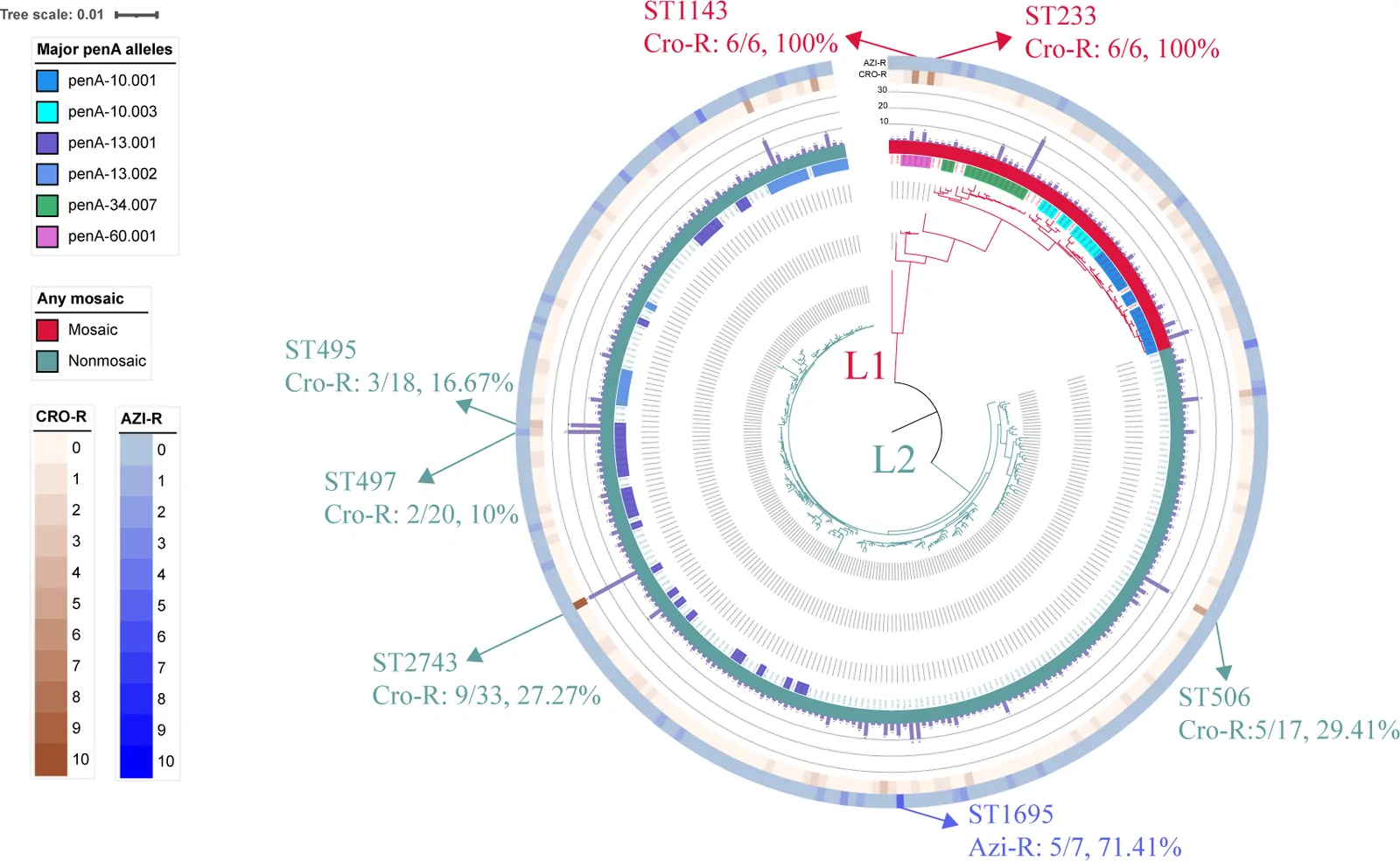
Phylogenetic tree of 281 NG-STAR STs constructed using MEGA 11. The external color trips that range from inner to outer are as follows: trip 1, mosaic or nonmosaic *penA* allele, crimson, mosaic *penA*, cadet blue, nonmosaic *penA*; trip 2, bar chart showing number of isolates in each ST; trip 3, number of Cro-R isolates in each ST; trip 4, number of Azi-R isolates in each ST. STs indexed in the main text were indicated with arrows outside trips.

Typing for AMR using the NG-STAR scheme identified 281 NG-STAR types with 154 new STs (Fig. S2B). The most common (top five) STs were ST2473 (33/664, 4.97%), ST2477 (23/664, 3.46%), ST497 (20/664, 3.01%), ST495 (18/664, 2.71%), and ST506 (17/664, 2.56%) (Fig. S2B). ST2473 and ST497 were also predominant STs in the surveillance database for 2014–2018 (10). Notably, the Cro-R rate of the “old” predominant ST2473 and ST497 significantly increased from 0.00% (0/42) in 2014–2018 to 27.27% (9/33) and 3.13% (1/32) in 2014–2018 to 10% (2/20) in 2019–2020, respectively, which may indicate that these clones rely on the ability to improve MICs to resist strong selection pressure (Fig. 3). Besides, ST495 and ST506 showed a notable Cro-R rate of 16.67% (3/18) and 29.41% (5/17), respectively (Fig. 3). Consequently, consistent with the phenotypic analysis, the AMR rates of most antimicrobials increased by varying degrees during 2019–2020, which may result in additional recombination of AMR alleles produced in *N. gonorrhoeae* to deal with selective pressure (19). The distribution of different ST types from 2019 to 2020 is listed in Table 1.

The phylogenetic analysis based on the seven AMR determinants showed that all 281 STs were grouped into two main clusters: L1 contains 59 STs (*n =* 143) that harbor mosaic *penA* alleles and L2 contains 222 STs (*n =* 521) that harbor nonmosaic *penA* alleles, respectively (Fig. 3). The proportion of Cro-R isolates in mosaic clusters (37/143, 25.87%) was significantly higher than that in nonmosaic clusters (78/521, 14.97%) (chi-square test, *P* = 0.004). The ST1143 and ST233 harboring *penA*-60.001 allele showed Cro-R rate of 100% (Fig. 3). In addition, the proportion of Azi-R isolates in mosaic clusters (4/143, 2.80%) was significantly lower than that in nonmosaic clusters (41/521,7.87%) (chi-square test, *P* = 0.037). Notably, the ST1695 showed a high Azi-R rate of 71.41% (5/7) (Fig. 3).

### Characterization of AMR determinants and Cro-R isolates

There were 51 *penA* types among the 664 isolates analyzed, including 16 mosaic (*n =* 143, 21.54%) and 35 nonmosaic (*n* = 521, 78.46%) types. The most common mosaic *penA* allele was *penA*-34.007 (49/143, 34.27%), followed by *penA*-10.001 (44/143, 30.77%), *penA*-10.003 (18/143, 12.59%), and *penA*-60.001 (15/143, 10.49%). The proportion of isolates harboring mosaic *penA* alleles increased from 8.39% (13/155) in 2014 to 20.56% (81/394) in 2020 (Table 1). Along with this trend, the rate of Cro-R in the mosaic group increased from 0.00% (0/13) in 2014 to 41.98% (34/81) in 2020. The continuous increasing rate of mosaic *penA* is likely to facilitate the development of AMR in gonorrhea treatment. In particular, the mosaic *penA*-60.001 showed rapid growth from 0.60% (1/166) in 2017 to 3.55% (14/394) in 2020, which proves that FC428 is no longer sporadic in China. Additionally, 14.97% (78/521) of the isolates harboring nonmosaic *penA* showed resistance to ceftriaxone. The most prevalent nonmosaic *penA* types were *penA*-13.001 (*n =* 91) and *penA*-18.001 (*n =* 71), of which both clones were the predominant ST in China, suggesting that the AMR of the nonmosaic group mainly occurred in the local common clones (Table 1).

A total of 18 isolates with A311V alteration were identified, 15 of which were in the *penA*-60.001 allele with 1 in *penA*-214.001 and 2 in *penA*-195.001 allele. The latter two were novel mosaics in the database with Cro-R and cefixime-resistant phenotypes, which were reported for the first time in this study.

### Phylogenomic analysis

**Fig 4:**
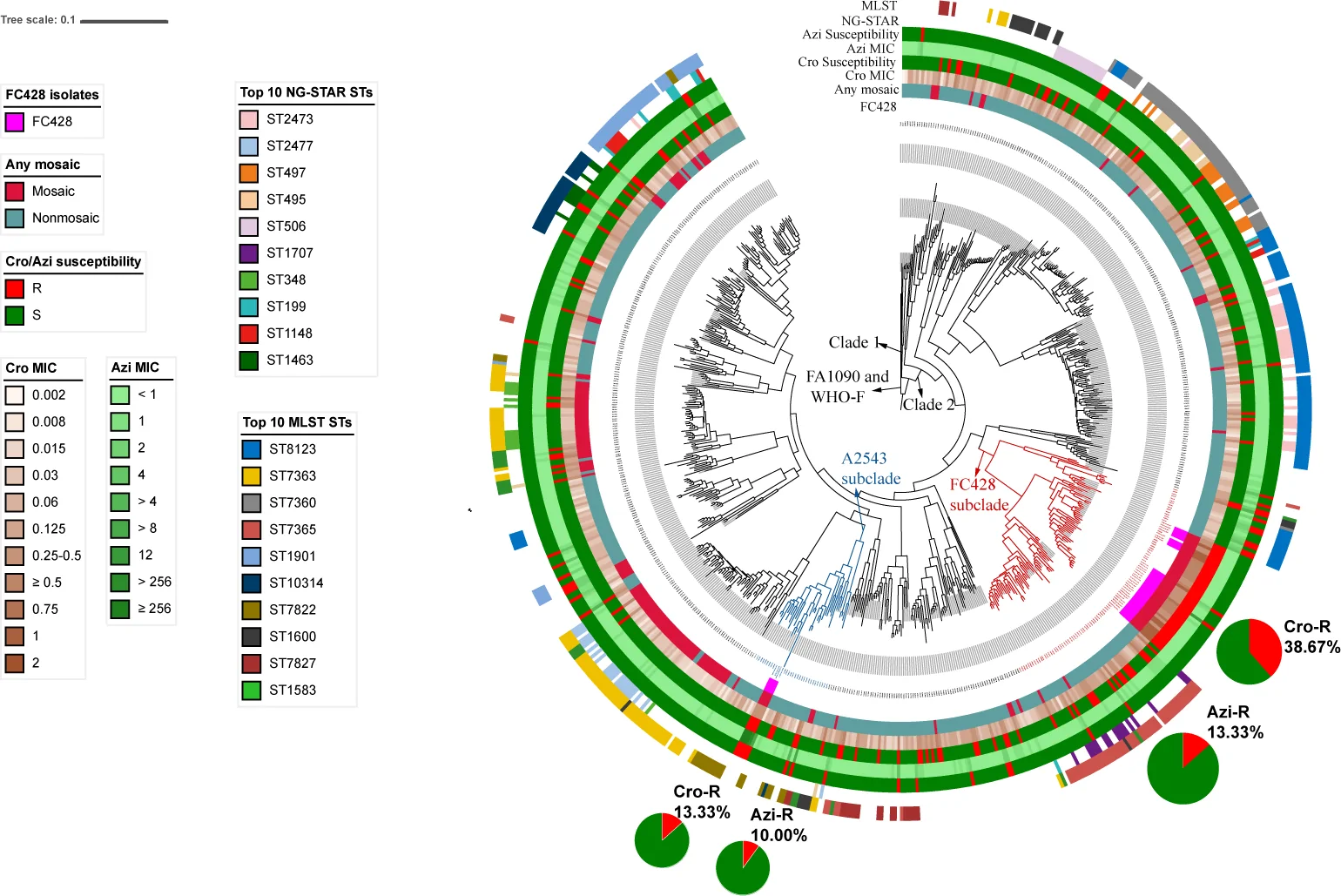
Maximum-likelihood phylogeny of 664 Shenzhen gonococcal isolates, 34 previously reported FC428 clones, and WHO-F. The maximum-likelihood phylogeny was constructed based on 46,305 SNP sites along the whole genome. The external color trips that range from inner to outer are as follows: trip 1, previously reported FC428 clones; trip 2, any mosaic *penA* alleles; trip 3, ceftriaxone MIC; trip 4, susceptibility categories of ceftriaxone (R, resistant; S, susceptible); trip 5, azithromycin MIC; trip 6, susceptibility categories of azithromycin (R, resistant; S, susceptible); trip 7, predominant NG-STAR STs; trip 8, predominant MLST STs. The percentages of Cro-R and Azi-R in FC428 subclade and A2543 subclade are represented by pie charts.

A maximum-likelihood phylogenetic analysis based on the core-genome alignment of 46305 SNP sites was performed. A total of 34 previously reported FC428 isolates and WHO-F were also mapped to FA1090 to construct a single-nucleotide variation phylogenetic tree. According to the ML phylogeny, the 698 gonococcal isolates (664 Shenzhen isolates plus 34 previously reported FC428 isolates) generated two distinct clades, namely, Clade 1 and Clade 2 (Fig. 4). Clade 1 represents that about 0.86% of the strains evolves earlier than Clade 2 that represents the rest of the strains (Fig. 4). Interestingly, in Clade 2, a total of 75 isolates from Shenzhen were observed closely related to 30 of the FC428 isolates and were clustered in the FC428 subclade (indicated in red in Fig. 4). Within the FC428 subclade, isolates from Shenzhen showed a high rate of Cro-R (38.67%, 29/75) as indicated in the pie charts (Fig. 4). Among the 29 Cro-R isolates in this subclade, 44.83% (13/29) and 51.72% (15/29) of the isolates harbored mosaic *penA*-60.001 and nonmosaic *penA*-13.002 allele, respectively. Besides, 48.28% (14/29) and 41.38% (12/29) of the Cro-R isolates were MLST ST7365 and ST1903, respectively. Both clones were closely related to ceftriaxone resistance among various surveillance, which was the main reason for the high proportion of ceftriaxone resistance in this clade (10, 20). Besides, in the FC428 subclade, 13.33% (10/75) isolates showed resistance to azithromycin, 60% (6/10) of which were the local predominant MLST ST8123.

In addition, 30 Shenzhen isolates were closely clustered in a Cro-R and high-level Azi-R A2543 subclade (A2543, A2735, AT159, and WHO-Q), which showed evidence of circulating in Asia (21) (indicated in blue in Fig. 4). Isolates in this subclade showed a 13.33% and 10.00% rate of ceftriaxone resistance and azithromycin resistance, respectively (Fig. 4). Over 93% of isolates in A2543 subclade harbored a nonmosaic structure, and MLST ST7822 presented the most predominant MLST type. The NG-STAR type distribution showed a high diversity.

## DISCUSSION

In this study, all antimicrobials, except for spectinomycin, showed varying levels of resistance since 2019. Ciprofloxacin and penicillin have been removed from the Chinese treatment guidelines for gonorrhea since 2007 (22); however, multidrug resistance was perpetuated at a high level (>70%) due to the high proportion (>50%) of prescription antimicrobials used in outpatient visits (23). Consequently, the repurposing of “old” antimicrobials is not applicable to the current situation in China (24). Fortunately, spectinomycin has been available since 2012 and might be considered as a potential option for the treatment of gonorrhea in Shenzhen. However, there is concern that resistance would be acquired rapidly once spectinomycin is introduced as a first-line monotherapy (2). Therefore, developing new antimicrobials and vaccines remains the most important interventions to curb widespread AMR of *N. gonorrhoeae*.

As predicted, such a substantial increase was found in the Cro-R group, from 0.00% in 2016 to 24.87% in 2020, which was mainly caused by the widespread and inappropriate use of ceftriaxone therapy (25). Notably, 5% or higher of Cro-R gonococcal isolates have been previously reported in Shanghai and Guangdong in China (26, 27), which indicates an alarming situation in which a period of untreatable gonorrhea may be near. To the best of our knowledge, this is the first report of a high rate of 24.87% ceftriaxone resistance. Besides, Shenzhen has unique demographic characteristics, such as large mobile population and immigrant population, and young permanent residents’ average age (15). This alarming situation highlights the need to enhance AMR monitoring in other countries because the AMR transmission around the world could be connected through travel (6). These studies should be focused on countries where ceftriaxone remains sufficient for most settings, such as Kyrgyzstan in Central Asia, a country near China, attached a perfect rate (100%) of susceptibility to ceftriaxone (28). This information reinforces the importance of the “comprehensive and specific monitoring” strategy, which is aimed at those countries that cannot afford the resources required for comprehensive surveillance, to nevertheless aim to monitor high-risk regions.

Among the 115 Cro-R isolates (115/664, 17.32%, Table 2), the isolates in mosaic group (37/143, 25.87%) showed a significant higher proportion than that in the nonmosaic group (78/521, 14.97%) (chi-square test, *P* = 0.004). However, an increase in nonmosaic *penA*-related Cro-R from 2014 (0/142, 0%) to 2020 (64/313, 20.45%) was observed, which strongly challenged the current strategies of molecular surveillance. The mosaic structure is widely recognized as a potential predictive marker for ceftriaxone resistance (29). The trend of increasing resistance rates in the nonmosaic *penA* group continues to weaken the effectiveness of current strategies and further reduce the efficacy of molecular epidemiology, which has been reported in several research (30, 31). In the Shenzhen region, the *penA*-13.002 (*n* = 15) and *penA*-60.001 (*n* = 15) showed the highest rate of Cro-R, and *penA*-13.002 is one of the most common local types in China, whereas it is rare in other countries, thereby decreasing the risk of global transmission (32). In addition, two novel mosaic *penA* types (*penA*-195.001, *n =* 2; *penA*-214.001, *n =* 1) were screened in this study and harbored *penA* A311V mutation, which is the well-known molecular marker to capture ceftriaxone-resistance-associated *penA*-60.001 (33). In addition, mutations in *mtrR*, *ponA*, and *porB* also provide contributions to resistance to beta-lactams (Table 2). Overall, the highly diverse mechanisms of ceftriaxone resistance pose a threat for the rapid detection of *penA*-60.001. This difficult situation highlights the importance of rapidly producing molecular targets for emerging resistant isolates based on basic science research.

**TABLE 2:** Detailed information of 115 Cro-R isolates in Shenzhen, China, 2019–2020

	Details
No. of isolates	115
No. of MLST STs	32
Predominant MLST STs	7365(15) 1903(12) 8123(12) 1583(8) 1901(8) 7360(7)
No. of NG-STAR STs	74
Predominant NG-STAR STs	2473(9) 233(6) 1143(6) 1696(5) 1707(5) 506(5)
No. of mosaic *penA* allele types	10
Predominant mosaic *penA* allele types	60.001(15) 10.001(12) 10.003(3)
No. of nonmosaic *penA* allele types	13
Predominant nonmosaic *penA* allele types	13.002(15) 13.001(13) 18.001(13) 12.001(10)
Predominant *mtrR* mutations	−35A Del(85) −35A Del, G45D(10)
Predominant *porB* mutations	G120K, A121D(51) G120K, A121G(32) G120K, A121N(14)
Predominant *ponA* mutations	L421P(113)

Compared to a previous surveillance in Shenzhen (10), no obvious change was observed in the distribution of the MLST genotype except for ST11231. The rate of ceftriaxone resistance of ST11231 increased from 0.00% (0/14) during 2014–2018 to 35.71% (5/14) during 2019–2020, and alarmingly, 40% (2/5) isolates showed a potential of multi-resistance (ceftriaxone resistance combined with Azi MIC value of 1 mg/L, exactly at the clinical breakpoint for resistance), which suggests that the potential threat of the local clone can evolve into dangerous resistance clones by acquiring new phenotypes within a short time. Another difference is that the cluster from the goeBURST analysis in this study was observed to be lower than that in previous surveillance, which suggests that the diversity of the genome complex tended to be stable during 2019–2020. Conversely, high diversity was observed in the distribution of the NG-STAR genotype. Among the top five STs in a previous surveillance in Shenzhen, only two STs (ST2473 and ST497) were still reported as the predominant epidemic clones (10). Both STs were recognized as ceftriaxone-resistance-related clones in a previous study (10), which may be the main reason for maintaining the stability of the two clones. Therefore, these resistance-associated STs (such as MLST ST11231, NG-STAR ST2473, and NG-STAR ST497) should be the focus of monitoring.

Whole-genome sequences provide a complete explanation of the characteristics of gonococcal isolates compared with those obtained from MLST and NG-STAR. In this study, a total of 644 NG isolates coupled with 34 previously described gonococcal isolates were performed phylogenetic analyses and generated two distinct clades, namely, Clade 1 and Clade 2 (Fig. 4). Specifically, there were 75 isolates clustered in the Cro-R FC428 subclade, which contained the internationally spreading FC428 isolates as well as some isolates from different regions of China (12, 34 – 36) (Fig. 4). In addition, 30 isolates were clustered with four Cro-R and high-level Azi-R isolates (A2543 subclade). Three of the four cases were associated with travel to Asia, suggesting a potential circulation in Asia (37 – 39) (Fig. 4). These results suggest that the FC428 clone was further disseminated locally and may further acquire azithromycin resistance along transmission. Furthermore, 10% of isolates clustered in the A2543 subclade showed azithromycin resistance (Fig. 4). It poses a risk of isolates in this subclade might acquire ceftriaxone resistance combined with high-level azithromycin resistance and threaten the dual-antimicrobial therapy, then bring burden to the management and control of gonorrhea on public health level.

Importantly, the global spread of the Cro-R *N. gonorrhoeae* FC428 isolates showed a typical increase from 0.60% (1/166) in 2017, first reported in Shenzhen, to 3.55% (14/394) in 2020. Notably, the FC428 was still reported sporadically in 2019 in the Shenzhen region, with explosive growth in just 1 year. Similar to China, the FC428 has been reported as an epidemic clone at 12.28% (14/114) in Vietnam during 2019–2020 (40), which proves that the FC428 has circulated in several countries. Consequently, countries should aim to monitor epidemiologically relevant FC428 clones, which are also the only clones that show 100% relevance to the resistance phenotype.

This study had several limitations. One was the uneven gender rate of samples because males have a higher ratio of outpatient visits. Another limitation was that most samples were collected from the symptomatic population because asymptomatic patients rarely choose to seek hospital care. The interpretability and comparability of our study may be influenced by these limitations, and we will further adjust the sampling strategy for future surveillance.

In conclusion, this study investigated the transmission pattern and evolution of AMR in the Shenzhen region between 2019 and 2020 and further characterized the resistance-associated epidemic clone. Our data provided real-time surveillance data to help match clinical guidelines to the actual patterns of AMR of *N. gonorrhoeae*. Overall, our findings provide current data on gonococcal AMR and are helpful for the adjustment and improvement of gonorrhea surveillance strategies.

## MATERIALS AND METHODS

### Antimicrobial susceptibility testing

All isolates perform species identification using culture method, and agar dilution methods were used to assess the isolates’ susceptibility to penicillin, ceftriaxone, cefixime, ciprofloxacin, tetracycline, azithromycin, and spectinomycin (10). The thresholds of resistance were defined in accordance with European Committee on Antimicrobial Susceptibility Testing (EUCAST) (https://www.eucast.org/clinical_breakpoints/): resistance to penicillin (Pen-R): MIC > 1 mg/L; resistance to ceftriaxone (Cro-R), MIC > 0.125 mg/L; resistance to cefixime (Cef-R), MIC > 0.125 mg/L; resistance to ciprofloxacin (Cip-R), MIC > 0.06 mg/L; resistance to tetracycline (Tet-R), MIC ＞ 1 mg/L; resistance to azithromycin (Azi-R), MIC > 1 mg/L; and resistance to spectinomycin (Spec-R), MIC > 64 mg/L.

### Genomic DNA extraction and WGS

Genomic DNA from each isolate was extracted and purified using a QIAamp DNA Mini Kit (Qiagen, Valencia, CA, USA) according to the manufacturer’s protocol. DNA libraries were prepared using a Nextera XT DNA Library Preparation Kit (Illumina, San Diego, CA, USA). Libraries were then sequenced using the Illumina NovaSeq 6000 platform, according to the manufacturer’s instructions.

### Quality control and phylogenic analysis

Trimmomatic software version 0.39 (http://www.usadellab.org/cms/?page=trimmomatic) was used to filter out the adapter sequence and low-quality bases/reads. A quality assessment of the sequence reads was performed using FastQC version 0.11.9 (https://www.bioinformatics.babraham.ac.uk/projects/fastqc/). After the QC procedures were completed, the clean reads were mapped to the reference strain FA1090 (GenBank accession no. AE004969.1) using BWA MEM (41).

Single-nucleotide polymorphisms (SNPs) were identified using VarScan version 2.4.3 (42) and SAMtools version 1.9 (43). SNPs within repetitive regions and prophages of the FA1090 genome, identified by RepeatMasker (http://repeatmasker.org/) and PHAST (44), were excluded. To avoid the potential effects of homoplasy of drug resistance-associated mutations in the phylogeny tree construction, SNPs located in known genes/regions, including *penA*, *porB*, *mtrR,* and *mtrR* promoter, were further excluded from the data set, as previously described (45). The SNP set obtained in the previous steps was used to construct the maximum-likelihood phylogeny using FastTree 2.1.10 (46). Sequencing data of all isolates were deposited in Sequence Read Archive (PRJNA560592).

### Molecular characterization and genotyping using MLST and NG-STAR

MLST and NG-STAR were performed using gene sequences extracted *in silico* from the WGS data by mapping clean reads to the reference genome FA1090 (GenBank accession no. AE004969.1) and submitted to the *Neisseria* MLST (http://www.mlst.net/) and NG-STAR (https://ngstar.canada.ca/welcome/home) websites to determine the respective STs. The allelic profiles for MLST were visualized using PHYLOViZ 2.0 to investigate the evolutionary patterns and explore the founding genotypes (10). A phylogenetic analysis was also performed using the seven AMR determinants of NG-STAR and a neighbor-joining phylogenetic tree with 1,000 bootstrapped replicates using MEGA 11 (10). The iTOL online tool was used for further phylogenetic tree visualization and annotation (10).

### Statistical analysis

Statistical analysis was performed using SPSS version 26.0 (Chicago, Illinois) for Windows. The chi-square test was used in this study, and a *P*-value of < 0.05 was statistically significant.

## SUPPLEMENTAL MATERIAL

Tables S1 to S2, Fig. S1 to S2 spectrum.01728-23-s0001.docx Demographic characteristics, MLST and NG-STAR distribution.

ASM does not own the copyrights to Supplemental Material that may be linked to, or accessed through, an article. The authors have granted ASM a non-exclusive, world-wide license to publish the Supplemental Material files. Please contact the corresponding author directly for reuse.
