# Supplementary material for: Genomic epidemiology of Neisseria gonorrhoeae in Shenzhen, China, during 2019–2020: increased spread of ceftriaxone-resistant isolates brings insights for strengthening public health responses
Source: Microbiol Spectr. 2023 Sep 21;11(5):e01728-23. doi: 10.1128/spectrum.01728-23 (PMC10580820; doi:10.1128/spectrum.01728-23)
Supplement: Tables S1 to S2, Fig. S1 to S2 — Demographic characteristics, MLST and NG-STAR distribution. [file spectrum.01728-23-s0001.docx]

***Appendix Information***

**Appendix information**

Table S1. Demographic characteristics of 664 isolates.

Table S2. Basic information of six groups analyzed by goeBURST.

FIG S1 Barplot of ceftriaxone MIC distribution.

FIG S2 Circular barplot showing the distribution of frequency of MLST and NGSTAR STs.

Table S1. Demographic characteristics of 664 isolates.

|  | 2019 | | 2020 | | Total | |
| --- | --- | --- | --- | --- | --- | --- |
|  | No. of | Proportion | No. of | Proportion | No. of | Proportion |
| Sex | | | | | |  |
| Male | 241 | 89.26% | 350 | 88.83% | 591 | 89.01% |
| Female | 29 | 10.74% | 44 | 11.17% | 73 | 10.99% |
| Age | | | | | |  |
| Medium | 30 |  | 29 |  | 30 |  |
| Sexual Orientation | | | | | |  |
| Heterosexual | 268 | 99.26% | 388 | 98.48% | 656 | 98.80% |
| Homosexual | 1 | 0.37% | 1 | 0.25% | 2 | 0.30% |
| Bisexual | 0 | 0.00% | 0 | 0.00% | 0 | 0.00% |
| Unknown | 1 | 0.37% | 5 | 1.27% | 6 | 0.90% |
| Course | | | | | |  |
| Mean ± SD | 3.34±2.01 |  | 4.28±5.66 |  | 3.95±4.81 |  |
| Unknown | 3 |  | 0 |  | 0 |  |
| Reason for encounter | | | | | |  |
| Abnormal discharge | 242 | 89.63% | 304 | 77.16% | 546 |  |
| Other | 20 | 7.41% | 78 | 19.80% | 98 | 14.75% |
| Unknown | 8 | 2.96% | 12 | 3.05% | 20 | 3.01% |
| History of antibiotic treatment | | | | | |  |
| Yes | 5 | 1.85% | 12 | 3.05% | 17 | 2.56% |
| No | 263 | 97.41% | 376 | 95.43% | 639 | 96.23% |
| Unknown | 2 | 0.74% | 6 | 1.52% | 8 | 1.20% |
| History of gonorrhoea | | | | | |  |
| Yes | 4 | 1.48% | 5 | 1.27% | 9 | 1.36% |
| No | 265 | 98.15% | 383 | 97.21% | 648 | 97.59% |
| Unknown | 1 | 0.37% | 6 | 1.52% | 7 | 1.05% |

Note: Other includes dysuria, itching, dyspareunia, hypogastralgia, sore throat, the patient is asymptomatic but whose partner has symptoms, and routine checkup, etc.

Table S2. Basic information of six groups analyzed by goeBURST.

|  | STs | Isolates | Edges |
| --- | --- | --- | --- |
| Group 0 | 68 | 655 | 139 |
| Group 1 | 1 | 5 | 0 |
| Group 2 | 1 | 1 | 0 |
| Group 3 | 1 | 1 | 0 |
| Group 4 | 1 | 1 | 0 |
| Group 5 | 1 | 1 | 0 |


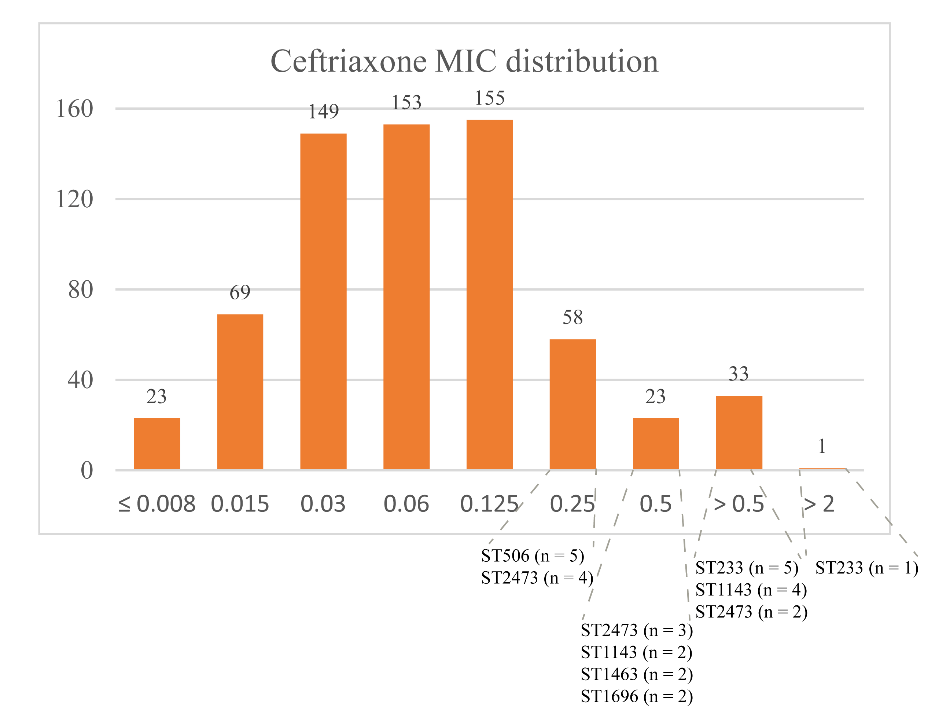


FIG S1 Barplot of ceftriaxone MIC distribution. X-axis: MIC value catalogues, Y-axis: number of isolates.


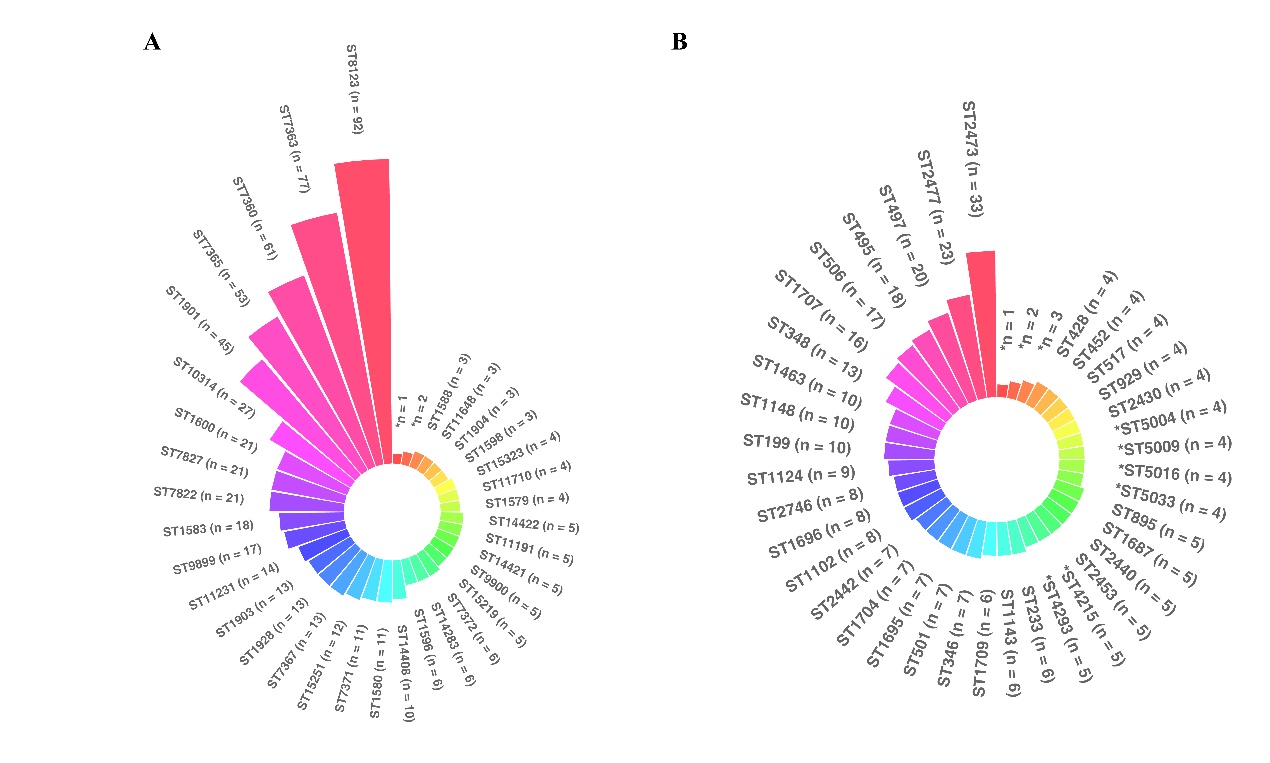


FIG S2 Circular barplot showing the distribution of frequency of MLST and NGSTAR STs. A. A total of 73 MLST ST types. B. A total of 281 NG-STAR ST types. STs with asterisks represents new STs identified in the current study.
